# Adipose tissue dysfunction in obese horses with equine metabolic syndrome

**DOI:** 10.1111/evj.13097

**Published:** 2019-04-10

**Authors:** A. Reynolds, J. A. Keen, T. Fordham, R. A. Morgan

**Affiliations:** ^1^ Royal (Dick) School of Veterinary Studies University of Edinburgh Roslin Midlothian UK; ^2^ University/BHF Centre for Cardiovascular Science the Queen's Medical Research Institute University of Edinburgh Edinburgh UK

**Keywords:** horse, adipose tissue, adipocyte, equine metabolic syndrome, obesity, insulin dysregulation

## Abstract

**Background:**

Obesity is a common feature of equine metabolic syndrome (EMS). In other species, obese adipose tissue shows pathological features such as adipocyte hypertrophy, fibrosis, inflammation and impaired insulin signalling all of which contribute to whole body insulin dysregulation. Such adipose tissue dysfunction has not been investigated in horses.

**Objectives:**

To determine if obese horses with EMS have adipose tissue dysfunction characterised by adipocyte hypertrophy, fibrosis, inflammation and altered insulin signalling.

**Study design:**

Cross‐sectional post‐mortem study.

**Methods:**

Samples of peri‐renal (visceral) and retroperitoneal adipose tissue were obtained at post‐mortem from healthy horses (n = 9) and horses with EMS (n = 6). Samples were analysed to determine average adipocyte size, fibrotic content and expression of inflammatory and insulin signalling genes.

**Results:**

Horses with metabolic syndrome showed marked adipocyte hypertrophy and increased expression of adipokines (leptin) and inflammatory cytokines (*TNFα*,*IL1β* and *CCL2*) in both adipose tissue depots compared to healthy horses. There were no differences in fibrosis or expression of genes relating to insulin signalling between the groups.

**Main limitations:**

Cases used in this study had advanced EMS and may represent the end stage of the condition; the design of the study is such that we were unable to relate the identified adipose tissue dysfunction to whole body insulin dysregulation.

**Conclusions:**

Horses with obesity and EMS have significant dysfunction of the peri‐renal and retroperitoneal adipose tissue that may contribute to whole body insulin dysregulation.

## Introduction

Equine metabolic syndrome (EMS), defined as a collection of risk factors including insulin dysregulation, genetic predisposition and obesity which increase the animal's susceptibility to laminitis, is common in domesticated horses 1. The increased risk of laminitis conferred by the presence of EMS makes this condition a significant welfare problem 2. Obesity, defined as excessive fat accumulation which may impair health, is a key feature of EMS in the majority of reported cases, but little is known about the pathology of obese adipose tissue in EMS.

Adipose tissue expands in response to calorie intake in excess of nutritional requirements either by increasing adipocyte number (hyperplasia) or increasing adipocyte size (hypertrophy) 3. In humans and dogs, hypertrophy of adipocytes is associated with whole body insulin resistance and elevated fasting plasma insulin 4, 5, whilst hyperplasia appears to be a protective phenotype associated with better insulin sensitivity and lipid profiles 6. Human obese adipose tissue is also characterised by increased fibrosis 7, infiltration of macrophages and a pro‐inflammatory phenotype 8, 9. The adipose tissue can itself become resistant to the effects of insulin but probably more significantly obese adipose tissue can contribute to whole body insulin resistance though the release of free fatty acids, pro‐inflammatory cytokines and adipokines 10.

Obese adipose tissue in horses also shows signs of dysfunction including signs of impaired mitochondrial function 11, altered insulin signalling 11, enhanced glucocorticoid metabolism 12. Other indications of adipose tissue dysfunction in obese horses include an altered plasma lipidome 13 and elevated plasma leptin concentrations 14. A pro‐inflammatory phenotype is not consistently reported in adipose tissue of horses with obesity, insulin dysregulation or EMS 15, 16, 17 though correlations between circulating inflammatory cytokines and adiposity are reported 16, 18, 19.

In this study, we aimed to further investigate obese adipose tissue in the horse. We focus on intra‐abdominal adipose tissue depots which are associated with the most pathological changes and associated morbidities in other species 20 and which expand rapidly in horses with obesity. We hypothesised that obese horses with EMS have visceral and retroperitoneal adipose tissue dysfunction characterised by adipocyte hypertrophy, fibrosis, inflammation and altered insulin signalling.

## Materials and methods

### Horses

Healthy horses and horses with EMS, destined for euthanasia, were recruited from clinics at the Royal (Dick) School of Veterinary Studies. Healthy horses were defined as those with no clinical (body condition score <4/5, no history or evidence of laminitis), histological (pituitary score <3/5) or biochemical evidence of endocrine disease (fasting basal insulin <20 mIU/L, plasma ACTH within the seasonal reference range) and no history of glucocorticoid administration within the previous 3 months. Horse with EMS were defined as those >2 years of age of a susceptible breed (native British breeds) with a body condition score ≥4/5 21, fasting basal insulin >20 mIU/L 1, current or historical laminitis, plasma ACTH within the seasonal reference range 22 and a pituitary histological score <3/5 23. ACTH and insulin concentrations were measured by chemiluminescent immunoassays (Immulite 2000)^a^. Horses were humanely subjected to euthanasia with quinalbarbitone sodium and cinchocaine hydrochloride (1 mL/10 kg bodyweight; Somulose)^b^. Samples of peri‐renal and retroperitoneal (lying deep to the linea alba) adipose tissue were collected and bisected, half was fixed in 10% paraformaldehyde overnight and half snap frozen and stored at −80°C.

### Adipocyte size

Five‐micrometre sections of the adipose tissue were stained with haematoxylin/eosin. Four randomly selected images of each slide were obtained (Olympus BX51) at 4× magnification. The images were validated manually and a minimum of three were analysed using Image J software with the Adiposoft v1.13 plugin 24 to determine cell area, the average of all the images was taken to given the final average adipocyte area (μm^2^).

### Adipose fibrosis

Sections (5 μm) of adipose tissue were stained for collagen with Picrosirius red (PSR). Whole slide scanning was performed at 5× viewing magnification using a Zeiss Axio Scanner^c^. Scanned images were manually checked. Staining was analysed using Fiji imaging software (Image J). Total fibrosis was expressed as the ratio of stained area to total tissue surface as previously described 25.

### Gene expression

Adipose tissue was homogenised in Qiazol^d^. Total RNA was extracted using the RNAeasy Mini Kit^e^. RNA quality was assessed using a Nanodrop Spectrometer. Five hundred‐nanogram of RNA was reverse transcribed using Quantitect Reverse Transcription Kit^f^ to synthesise cDNA.

Quantitative real‐time polymerase chain reaction was performed using a Light‐cycler 480^g^. The most appropriate housekeeping genes for each tissue were determined by testing six candidates and using NormFinder software (MOMA, Arhaus University Hospital, Denmark) 26; for both depots these were 18s and succinate dehydrogenase (SDHA). Target gene expression was arbitrarily quantified against a standard curve constructed from pooled samples for each primer probe combination corrected by two housekeeping genes. Expression of genes associated with fibrosis (collagen 1α, hypoxia inducible factor 1α, smooth muscle actin), macrophage recruitment (chemokine [C‐C motif] ligand 2, also referred to as monocyte chemoattractant protein 1) inflammation (tumour necrosis factor α, interleukin 1β), adipokine release (leptin, adiponectin) and insulin signalling (insulin receptor, insulin receptor substrate 1, mammalian target of rapamycin, mTOR) was analysed. See Supplementary Item 1 for primer sequences.

### Data analysis

Based on preliminary data, a power calculation determined that five horses per group would give 85% power to detect a 50% increase in average adipocyte size (healthy 5000 μm^2^ ± 2500 vs. EMS 7500 μm^2^ ± 2500). Statistical analysis was performed using Graph Pad Prism 5^h^ and SPSS statistics 19^i^. Data were tested for normality using a Kolmogorov‐Smirnov test. Data from the two groups were analysed using a Student's *t* test or Mann‐Whitney test as appropriate. Data were also analysed by two‐way ANOVA for the effect of adipose tissue depot. Significance was set at P<0.05. Data are presented as mean (±s.e.).

## Results

Nine healthy horses and six horses with EMS were recruited to the study. Table 1 shows their clinical characteristics.

**Table 1:** Clinical and biochemical characteristics of the study groups

	Healthy (n = 9)	EMS (n = 6)	P value
Age (years)	17.4 ± 2.3	18.8 ± 1.6	0.1
Breed	4 Thoroughbreds, 2 Welsh Cob X Thoroughbred, 2 Irish Sports Horses, 1 Trakhener X Thoroughbred	4 Welsh cobs, 1 Shetland, 1 Icelandic Horse)	
Body condition score (/5)^a^	2 (IQR 1.75–2.0)	4.5 (IQR 4–5)	<0.001
Fasting insulin (mIU/L)	7.1 ± 4.5	51.4 ± 15.8	<0.001
ACTH (pg/mL)	34.3 ± 4.3	34.0 ± 5.7	0.9

Data are mean ± s.e.

a The body condition score (BCS, /5) as a measure of adiposity (Carroll and Huntington 21), Comparisons between groups were by Student's *T* test or Mann‐Whitney *U* test. *P<0.05 vs. healthy group.

### Adipocyte size

**Figure 1:**
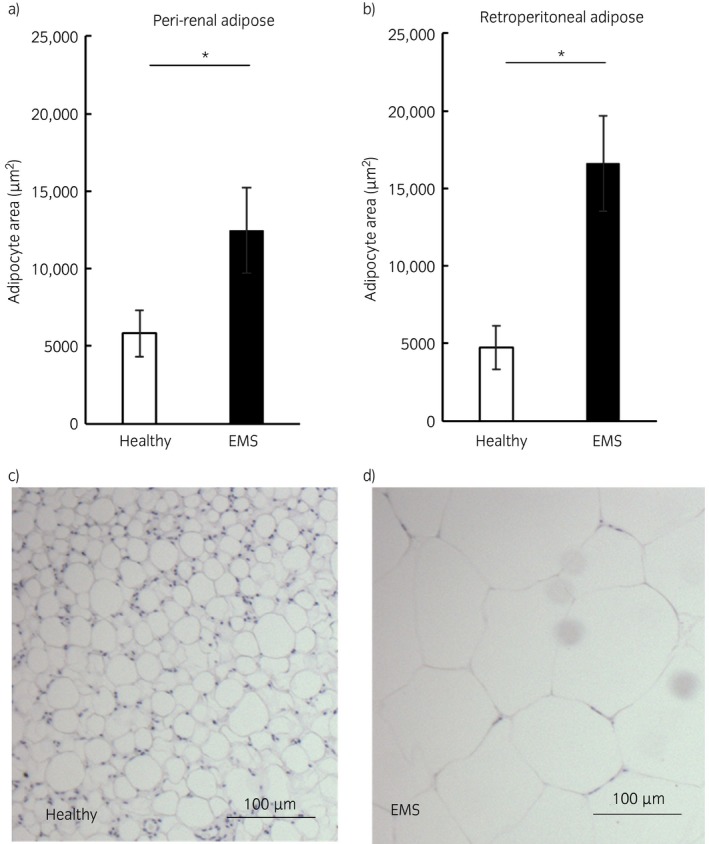
Mean adipocyte area in peri‐renal and retroperitoneal adipose tissue of healthy horses and horses with equine metabolic syndrome (EMS). Graphs showing the mean (±s.e.) adipocyte area (μm^2^) in peri‐renal a) and retroperitoneal b) adipose tissue of healthy horses and horses with EMS. *P<0.05. Representative micrographs of peri‐renal adipose from a healthy horse c) and a horse with EMS d) (×20 magnification). Adipocytes were significantly larger in the peri‐renal and retroperitoneal depots of horses with EMS compared with healthy controls.

Adipocytes from horses with EMS from both the peri‐renal (healthy 5818 ± 1494 vs. EMS 12481 ± 2758 μm^2^ P = 0.004) and retroperitoneal depots (healthy 4751 ± 1406 vs. EMS 16621 ± 3082 μm^2^, P = 0.002) were significantly larger than those from healthy horses (Fig 1). Adipose tissue depot had no significant effect on adipocyte size.

### Adipose fibrosis

**Figure 2:**
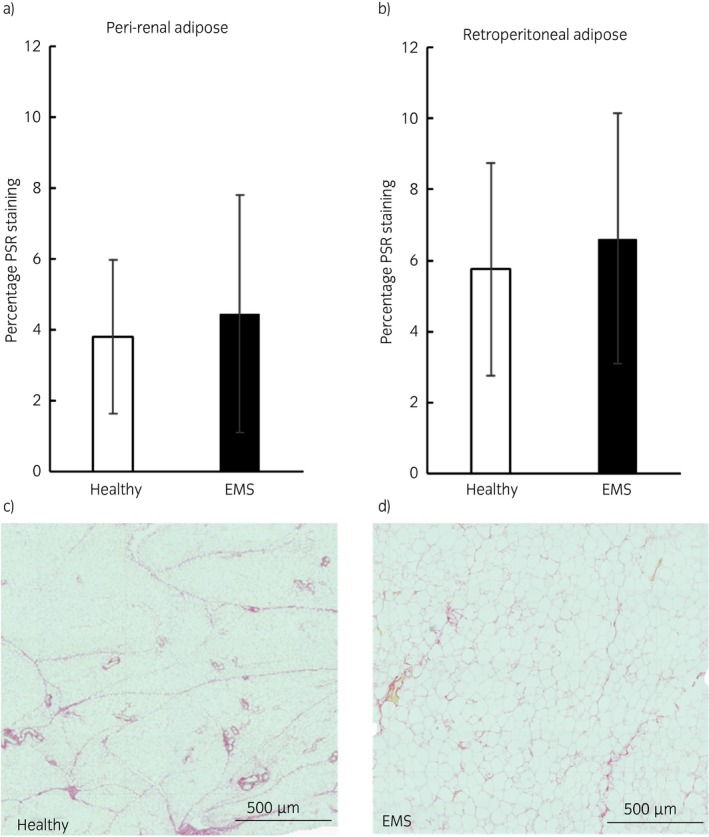
Adipose fibrosis in peri‐renal and retroperitoneal adipose tissue of healthy horses and horses with equine metabolic syndrome (EMS). Graphs showing the mean (±s.e.) picrosirius red (PSR) staining expressed as a percentage of the total section area in peri‐renal a) and retroperitoneal b) adipose tissue from healthy horses and horses with EMS. Representative micrographs of peri‐renal adipose from a healthy horse c) and a horse with EMS d) showing PSR staining (×5 magnification). There were no differences in the percentage of PSR staining of the peri‐renal or retroperitoneal adipose depots between healthy horses and those with EMS.

Picrosirius red staining was not significantly different between healthy horses and horses with EMS (Fig 2). Adipose tissue depot had no significant effect on the quantity of staining.

### Adipose tissue gene expression

#### Adipokines

**Figure 3:**
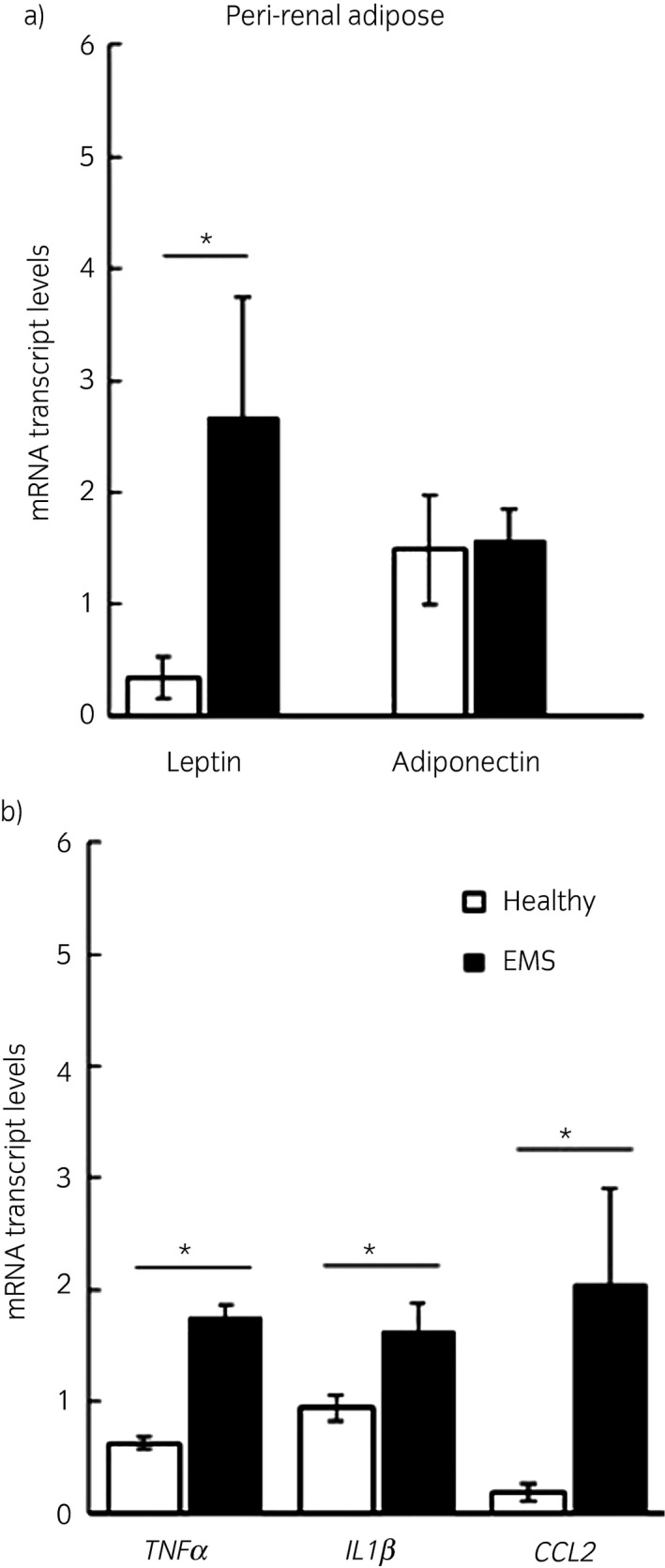
Peri‐renal adipose gene expression in healthy horses and horses with EMS. Graphs showing peri‐renal adipose mRNA transcript levels of adipokines, leptin and adiponectin (a) and inflammatory cytokines tumour necrosis factor α (*TNFα*), interleukin 1β (*IL1β*) and chemokine (C‐C motif) ligand 2 (*CCL2*). (b) Transcript levels are expressed as a ratio to housekeeping genes 18s and succinate dehydrogenase. *P<0.05.

**Figure 4:**
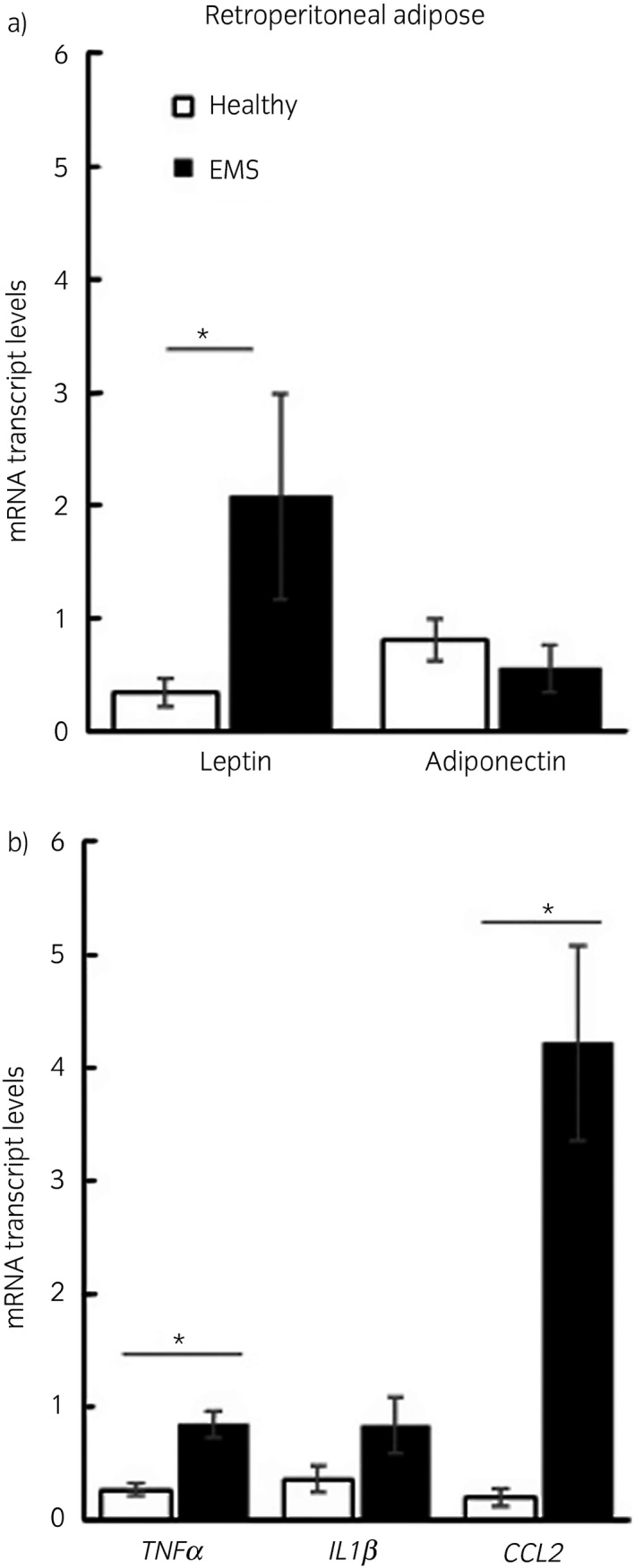
Retroperitoneal adipose gene expression in healthy horses and horses with equine metabolic syndrome. Graphs showing retroperitoneal adipose mRNA transcript levels of adipokines, leptin and adiponectin (a) and inflammatory cytokines tumour necrosis factor α (*TNFα*), interleukin 1β (*IL1β*) and chemokine (C‐C motif) ligand 2 (*CCL2*). (b) Transcript levels are expressed as a ratio to housekeeping genes 18s and succinate dehydrogenase. *P<0.05.

Leptin expression was significantly higher in both peri‐renal (P = 0.01) and retroperitoneal (P = 0.005) adipose tissue from horses with EMS compared with healthy horses. Adiponectin expression was not different between the groups in either depot (Figs 3, 4). Adipose tissue depot had no significant effect on expression.

### Adipose tissue morphology

There was no difference in expression of collagen 1α, the predominant component of fibrotic tissue or *HIF1α*, an inducer of fibrosis, between the groups in either depot. Blood vessel marker smooth muscle actin expression was also not different between the groups in either depot (Supplementary Items 2 and 3). Adipose tissue depot had no significant effect on expression.

### Insulin signalling

None of the candidate genes related to insulin signalling (*IR*,* IRS1* and *mTOR*) were differentially expressed between the groups in either adipose tissue depot (Supplementary Items 2 and 3). Adipose tissue depot had no significant effect on expression.

### Inflammation

Expression of inflammatory cytokines *TNFα* (P = 0.001) and IL1β (P = 0.01) was higher in peri‐renal adipose tissue from horses with EMS compared with healthy horses. *TNFα* (P = 0.008) but not *IL1β* (P = 0.06) was higher in retroperitoneal adipose tissue from horses with EMS compared with healthy horses. *CCL2*, a monocyte chemoattractant, was significantly higher in both depots (peri‐renal P = 0.007, retroperitoneal P = 0.02) in horses with EMS compared with healthy horses (Figs 3, 4). Adipose tissue depot had no significant effect on expression.

## Discussion

In this study, we hypothesised that adipose tissue from horses with metabolic syndrome would be dysfunctional with adipocyte hypertrophy, fibrosis, inflammation and altered insulin signalling. Our data demonstrate that adipocyte hypertrophy, inflammation and increased *CCL2* expression are features of obese adipose tissue in horses but that fibrosis and altered adipose tissue expression of insulin signalling genes are not consistent features.

Increased adipocyte size, hypertrophy, in response to excess calorie intake is associated with insulin resistance and dyslipidaemia in humans whereas increasing adipocyte number, hyperplasia, is associated with a favourable metabolic profile 3. Hypertrophy occurs when there is an imbalance between lipid storage and lipolysis. Defects in lipolysis can occur secondary to insulin resistance but equally engorgement of adipocytes with excess lipid leads to dysfunction of the adipose tissue which can contribute to whole body insulin dysregulation for example through release of free fatty acids, adipokines and cytokines 10. In a canine model of induced obesity, adipocyte size in the visceral depot was a strong predictor of whole body insulin resistance and the authors concluded that hypertrophy developed first and played a key role in the development of insulin resistance 5. In our study, we demonstrate that obese horses with EMS display marked adipocyte hypertrophy and this is accompanied by inflammation. The horses in this study have resting hyperinsulinaemia, but we cannot say for certain whether the hypertrophic nature of the adipose tissue is a cause or a consequence of insulin dysregulation in these animals. Equally, we have not established whether obese horses always have hypertrophy or whether it is only hypertrophied when associated with insulin dysregulation.

While circulating inflammatory cytokines are fairly consistently elevated in horses with obesity and/or insulin dysregulation, studies of adipose tissue have generally been less consistent, though most studies have used subcutaneous depots 15, 16, 17, 18. Similar to other species 27, adipocyte hypertrophy in horses in this study was accompanied by increased expression of the adipokine leptin and the inflammatory cytokines *TNFα*,* IL1β* and *CCL2*. *CCL2*, also known as monocyte chemoattractant protein‐1, mediates macrophage infiltration into adipose tissue 28. Obesity in humans and mice is known to induce a pro‐inflammatory phenotype in resident adipose tissue macrophages as well as inducing macrophage recruitment 29. Macrophages are the main source of TNFα and IL1β. TNFα in particular is known to induce changes within adipocytes which have harmful effects on whole body insulin signalling; for example, TNFα increases lipolysis causing free fatty acid release, decreases peroxisome proliferator activated receptor‐gamma (PPARγ) expression and has direct actions on muscle insulin signalling 30. As such macrophages have been identified as a therapeutic target in treating insulin resistance in humans 31 and our data suggest they may also be important in EMS.

Leptin gene expression was also markedly increased in the adipose tissue of horses with EMS, a finding which is in agreement with other studies reporting increased plasma leptin concentrations in EMS and in induced obesity 13, 32. Adipocyte volume is the predominant determinant of leptin expression in adipose tissue in other species 33 as such leptin may be a marker of adipocyte hypertrophy and not just adiposity per se. Adiponectin is also inversely proportional to adipocyte size but the relationship is not as well characterised as that of leptin 27; in our study there was no difference in adiponectin expression in obese adipose tissue from horses.

Increased fibrosis was not a significant feature in the depots we studied. While fibrosis of subcutaneous adipose tissue is associated with poor glucose control and deleterious lipid profiles in humans, fibrosis of omental adipose tissue appears to limit adipocyte hypertrophy and is associated with better glucose and lipid control 7. Less work has been carried out in other species though upregulation of extracellular matrix genes is reported in induced obesity in dogs 34. While our study was adequately powered to detect a difference in adipocyte size, a retrospective power calculation shows that it was insufficiently powered to detect subtle differences in PSR staining due to the variability within the groups (38 horses per group would be required to give 85% power to detect a difference in groups; healthy 3.79% ± 2.17% vs. EMS 4.46% ± 3.35%); despite this it seems clear that hypertrophy and not fibrosis is the hallmark feature of obese adipose tissue in horses.

The relationship between adipose tissue dysfunction and insulin resistance is a complex and poorly understood one. Adipose tissue itself can be insulin resistant but most now argue that the pathological changes to the adipose tissue which contribute to whole body insulin dysregulation such as adipokine and cytokine release are more significant 35. In this study, we did not identify any significant differences in expression of the candidate genes relating to insulin signalling that we chose to analyse. Marycz *et al*. 11 reported increased insulin receptor and IRS expression in subcutaneous adipose tissue of Polish warmbloods with EMS, our conflicting data may therefore be due to the different depots we chose to study. There are clear depot‐specific differences in insulin signalling and gene expression in humans and horses 36, 37, 38. It should be noted that this candidate gene approach for assessing insulin signalling is very limited given the vast number of genes and the dynamic nature of insulin signalling and it tells us little about function which is generally governed by phosphorylation events.

As with any clinical study, our data are from a diverse range of horses with varying degrees of disease the duration of which was not documented; as such we are limited in our ability to draw conclusions in terms of cause and effect. Further studies in models of induced equine obesity are necessary to further define the relationship between adipose tissue dysfunction and insulin dysregulation and critically define the time point in weight gain when adipose tissue becomes ‘unhealthy’. Our inclusion criteria dictated that those horses in the EMS group were genetically predisposed that is native breeds for example, Welsh ponies. As such the breed distribution was very different between the groups and we cannot rule out breed effects on adipocyte size and expression profile. However, human twin studies suggest that while the hyperplastic response to excess calorie intake is largely genetically determined the hypertrophic response is less so 39. There were no differences in age between the groups in our study, they were all mature. In other species, advancing age is associated with a reduction in adipocyte area and an increase in fibrosis 40, further work is required to understand the effects of age on equine adipose tissue. We only included horses that had obesity and EMS in this study, whilst this represents the majority of EMS cases, horses that are apparently lean but with insulin dysregulation are described 1, we cannot rule out inadvertent inclusion of such horses in our healthy control group.

Nuchal crest adipose and rump adipose tissue are often studied as subcutaneous adipose depots in horses 41; in our experience the fibrosis within the nuchal crest depot is so extensive that this masks adipocyte‐specific pathology and in lean horses getting a representative sample of adipose tissue is often technically challenging. Rump subcutaneous adipose tissue often contains muscle fibres and presents similar problems 17, as such we chose to focus on visceral and retroperitoneal depots which are associated with the most adverse effects in other species 20 and which expand rapidly in horses with obesity. We chose to use peri‐renal adipose tissue as our visceral depot of choice as it is consistently present even in very lean horses, study of omental, mesenteric and peri‐cardial adipose tissue depots would be a useful extension to our work.

In conclusion, we have demonstrated that visceral and retroperitoneal adipose tissue of horses with obesity and EMS is markedly dysfunctional with a hypertrophic‐inflammatory phenotype. In educating owners about EMS, it is important to emphasise the ‘unhealthy’ nature of obese adipose tissue and its effects on the rest of the body, particularly in potentially inducing or worsening whole body insulin dysregulation.

## Authors’ declaration of interests

No competing interests have been declared.

## Ethical animal research

This study was approved by the University of Edinburgh Veterinary Ethics and Research Committee (approval 7014).

## Owner informed consent

Informed consent was obtained from all owners.

## Sources of funding

Supported by a BBSRC/Pfizer CASE Studentship (Grant Number R42126/82976) and the Wellcome Trust (ISSF2). R. Morgan's Clinical Career Development Fellowship is funded by the Wellcome Trust (206587/Z/17/Z). The laboratory is supported by a British Heart Foundation Centre of Excellence Award.

## Authorship

A. Reynolds contributed to study execution, data analysis and preparation of the manuscript. J. Keen contributed to study design and sample collection. T. Fordham contributed to study execution. R. Morgan contributed to study design, data analysis and preparation of the manuscript. All authors gave their final approval of the manuscript.

## Supporting information


**Supplementary Item 1:** Details of primers used for real time PCR.

**Supplementary Item 2:** Peri‐renal adipose gene expression in healthy horses and horses with EMS (insulin signalling and adipose morphology genes).

**Supplementary Item 3:** Retroperitoneal adipose gene expression in healthy horses and horses with EMS (insulin signalling and adipose morphology genes).

## Manufacturers' addresses

1 Siemens, Camberley, Surrey, UK.

2 Dechra Veterinary Products, Shrewsbury, Shropshire, UK.

3 Carl Zeiss Microscopy GmbH, Jena, Germany.

4 QIAGEN, Hilden, Germany.

5 Qiagen Inc., Valencia, California, USA.

6 Invitrogen, Carlsbad, California, USA.

7 Roche Applied Science, Indianapolis, Indiana, USA.

8 GraphPad Software Inc., La Jolla, California, USA.

9 IBM Software, New York, USA.
