# Supplementary material for: Adipose tissue dysfunction in obese horses with equine metabolic syndrome
Source: Equine Vet J. 2019 Apr 10;51(6):760–6. doi: 10.1111/evj.13097 (PMC6850304; doi:10.1111/evj.13097)
Supplement: Supplementary file 1 — Supplementary Item 1: Details of primers used for real time PCR. [file EVJ-51-760-s001.pdf]

BEVA Equine Veterinary Journal

Supplementary Item 1: Details of primers used for real time PCR.

Gene	Left Primer (5'-3')	Right Primer (5'-3')
Ribosomal RNA 18s (*18s*)	tgacccaaggctagtagctga	ttcaacacatcacccaccat
Succinate dehydrogenase (*SDHA*)	ctacggagaccttaagcatctga	gggtctccaccaggtcagta
Collagen 1α (*Col1α*)	tccccaaggtttccaagg	accatcatctccgttcttgc
Smooth muscle actin (*SMA*)	cggccatgtatgtggctatt	ctccagagtccagcacaatg
Hypoxia inducible factor 1α (*HIF1α*)	gcaaagaatccattttccactc	tcatccattgggatgtaagga
Adiponectin (*Adipq*)	tgtggtctgattctacactgagg	actcggcaggactagtagcaa
Leptin (*Lep*)	ttgtcaccaggatcaatgaca	gtccaaaccagtgaccctct
Insulin Receptor substrate 1 (*IRS1*)	atgccagcatcagtttcca	ggatttgctgaggtcatttagg
Insulin receptor (*IR*)	cctgagtgtccctctgggta	caggcacggggtacacat
Mammalian target of rapamycin (*mTOR*)	tggaagagtgctcgcaaga	gctgcatggtctgaacaaag
Interleukin 1β (*IL-1β*)	cctgtcttgtgggatgaaaga	tgggtaagtattggggtctactg
Tumour necrosis factor α (*TNFα*)	cttctgcctgctgcacttt	gactggaaggcattcggtaa
Chemokine (C-C motif) ligand 2 (*CCL2*)	cctccagcatgaaggtctct	gcagcaggtgactggagaat
