# Supplementary material for: Adipose tissue dysfunction in obese horses with equine metabolic syndrome
Source: Equine Vet J. 2019 Apr 10;51(6):760–6. doi: 10.1111/evj.13097 (PMC6850304; doi:10.1111/evj.13097)

BEVA Equine Veterinary Journal

Supplementary Item 2: Peri-renal adipose gene expression in healthy horses and horses with EMS (insulin signalling and adipose morphology genes).

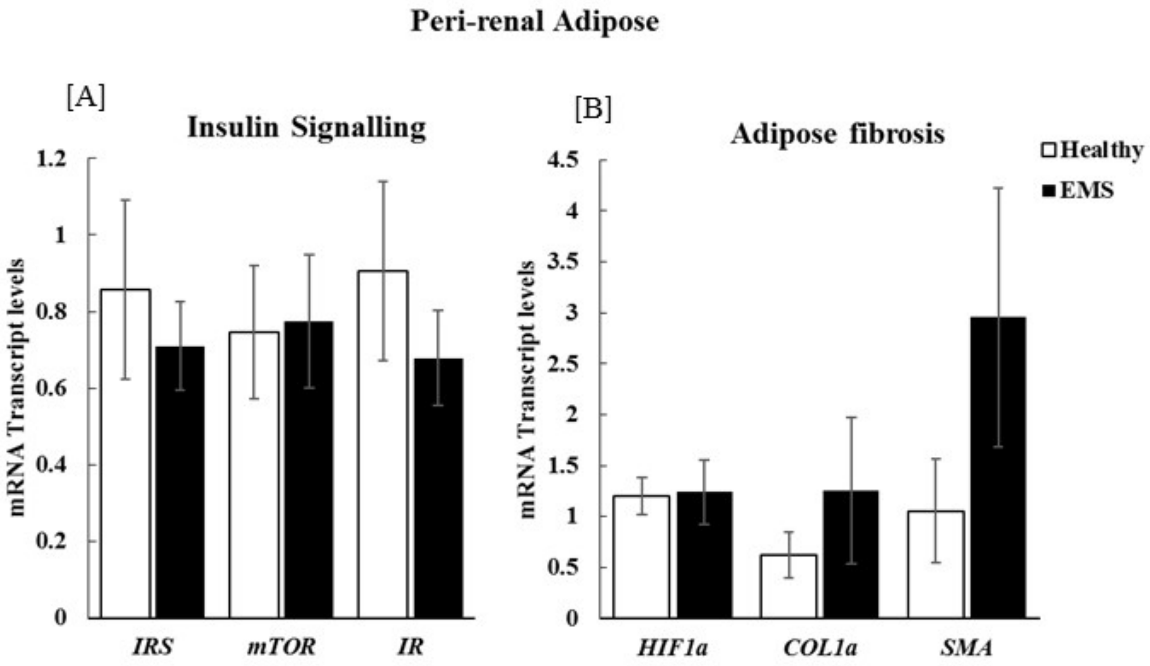

Supplement: Supplementary file 2 — Supplementary Item 2: Peri‐renal adipose gene expression in healthy horses and horses with EMS (insulin signalling and adipose morphology genes). [file EVJ-51-760-s002.pdf]
